# The Role of Artificial Intelligence and Machine Learning in Polymer Characterization: Emerging Trends and Perspectives

**DOI:** 10.1007/s10337-025-04406-7

**Published:** 2025-04-04

**Authors:** Rick S. van den Hurk, Bob W. J. Pirok, Tijmen S. Bos

**Affiliations:** 1 Analytical Chemistry Group, Van’t Hoff Institute for Molecular Sciences, University of Amsterdam, Amsterdam, The Netherlands; 2 Centre for Analytical Sciences Amsterdam (CASA), Amsterdam, The Netherlands

**Keywords:** Polymer characterization, Machine learning, Artificial intelligence

## Abstract

The application of artificial intelligence (AI) and machine learning (ML) is rapidly expanding and has begun to make a significant impact on polymer development and characterization. This perspective article explores the current state of AI in this field and highlights areas where its potential remains underutilized. While the optimization of polymer synthesis to achieve desired properties and the classification of polymer types are well-established, opportunities for AI integration in detailed characterization, analytical method development, and data processing remain largely untapped. Greater automation of the analytical laboratory, whether through dedicated algorithms or AI-driven solutions, will enable analytical chemists to focus more on addressing research questions and interpreting results, rather than on method development and routine measurements.

## Introduction

In modern society, polymers play a pivotal role, with applications spanning everyday items (e.g., consumer electronics, photovoltaics, coatings, food packaging, etc.) to advanced materials (e.g., medical implants, space travel). To address present and future challenges, the development of sophisticated polymers with increasingly tailored properties is essential. While polymer synthesis offers significant flexibility in achieving diverse properties, chemists ultimately require reliable information to optimize the synthesis process. Generally, this information can be obtained through analytical methods, or physical property-based relations combined with trial-and-error experiments. Often, the physical properties of a polymer can be linked to its molecular structure, offering valuable insights into the structure–property relationship.

To streamline the research and development process and provide this information on the structure–property relationship, automated platforms have emerged as valuable tools for polymer synthesis [1–4]. The advent of machine learning (ML) has only contributed to this for cases where optimal polymer properties can be represented in a scoring framework. This score quantifies the desirability or the optimality of the polymer properties and can then be used to guide ML methods to facilitate systematic exploration, effectively automating the trial-and-error process [5–7]. Integrating chemical information into such ML models further enhances their predictive capabilities, transforming the process into a more informed and efficient endeavor. This chemical information can be obtained through detailed simulations or advanced characterization techniques. Beyond refining the methods themselves, chemometrics and ML also play critical roles in the optimization of the analytical methods [8] as well as in analyzing and interpreting the results of these methods [9].

Unfortunately, despite the availability of numerous publications and reviews highlighting the promise of machine learning in the polymer field [1, 4–7, 9], its practical application remains limited in several key areas.

In this perspective article, we examine the current applications and future potential of artificial intelligence (AI), particularly ML, in advancing polymer science and characterization. We first clarify the terms AI and ML and then review a select number of areas in which ML has been demonstrated to deliver on the great promise. The reviewed literature is then used to recalibrate expectations toward future outlook.

## Definitions of Machine Learning and Artificial Intelligence

It is essential to first clarify the term AI as it is often misapplied to any automated workflow or algorithm. AI specifically refers to algorithms or robots that are capable of mimicking and surpassing human capabilities by perceiving and interacting with an environment [10]. In the subset of AI known as ML, a common approach involves providing solutions based on a set of parameters either directly or iteratively. The outcomes are evaluated through one or more scores, and depending on the algorithm, it either seeks to maximize these scores or predict them based on parameter combinations. A relevant example is an ML algorithm designed to optimize specific or various properties of a polymer by iteratively refining the synthesis process based on feedback [11].

## Machine Learning to Aid Polymer Development

### Simulation of Properties of Polymers

An effective initial approach to identify which chemical properties influence the physical properties of a final polymer product is to utilize simulations. While polymer simulations often do not fully replicate real-world conditions, they provide valuable insights into identifying aspects of interest. For both simulation-derived and experimentally obtained data, it is crucial to describe structural information in an organized and standardized manner.

One framework that facilitates this standardization is Polydat, which allows for the recording of both structural data and characterized parameters [12]. Such efforts toward standardization can greatly benefit the polymer characterization community, enabling models to more readily integrate and utilize data from other researchers. Commonly, general polymer structures are reported using BigSMILES notation [13–16], an extension of the normal SMILES format that incorporates features specific to polymers, such as repeating units, branching, and end groups. However, BigSMILES representations are often too complex for direct use in the training of models. To address this, molecular descriptors can be employed to simplify the structural information [17]. This reduction in parameter space not only streamlines model development but also helps generalize the features responsible for the properties of interest. This standardization effort helps in the creation of a database that enables the research community to collaborate to build more advanced models [18, 19]. Once polymers are accurately described, ML models can be trained to predict their properties based on composition and/or structural information [11].

### Optimization of Properties

ML proves highly effective for optimizing measurable properties and modifying synthesis conditions to enhance those and potentially other related properties [20, 21]. Notable examples of such applications include the design of polymer-based biomaterials [22], polymeric long-acting injectables [23], and orodispersible films used for drug delivery [24]. To perform these optimizations, it is first necessary to define the features of interest (polymer properties) and the adjustable variables [25]. The number of variables that can be adjusted simultaneously is often constrained as the search space expands exponentially with each additional variable. Features of interest can be selected either manually by the analyst or automatically using tools such as principal component analysis (PCA). Following the optimization process, interpreting the impact of various features is highly valuable. Eliminating features with minimal impact streamlines the workflow while the insights gained contribute to a deeper understanding of the underlying mechanisms [26].

### Polymer Synthesis and Discovery

As described in the previous section, the synthesis conditions may be modified to achieve more desirable polymers. Some works have developed closed-loop automated workflows to achieve this while incorporating ML, flow chemistry synthesis, and automated chemical analysis. The use of flow chemistry for optimizing synthesis has been demonstrated for multiple applications, including optimizing the yield for photocatalysis [27], and the optimization of functionality of various polymers [28]. For polymer synthesis, a property of interest may be the monomer conversion where ideally 100% of the monomers are converted. To achieve this, a flow reactor was connected with nuclear magnetic resonance (NMR) [29]. Moreover, it can be coupled to size-exclusion chromatography (SEC) to assess the molar mass dispersity as a second parameter [30]. The closed-loop system automatically processes the SEC and NMR data to obtain the dispersity and monomer conversion. This data was fed into a Thompson sampling efficient multi-objective optimization (TS-EMO) model with every iteration to predict and subsequently identify the Pareto front for these objectives. It should be noted that this workflow can only identify the Pareto optimum within the user-defined reaction space (e.g., a maximum residence time of 20 min and a temperature range of 80–120 °C). While the previous synthetic routes were performed in flow, some reactions are better suited for batch processes. As such, a similar automated setup may be designed that enables batch operation modes [31]. It has, however, not yet been combined with online characterization and ML algorithms for the prediction of polymer properties.

Besides optimizing synthesis parameters, a similar system can be used to discover better-performing copolymers. Using a similar TS-EMO-based algorithm, conflicting optimization objectives, such as cost and yield, may be optimized using inline reversed-phase LC (RPLC) to determine yield for a single-step synthesis [32]. Automated flow synthesis has been used to discover ^19^F magnetic resonance imaging agents by coupling it directly with ^19^F NMR analysis [33]. Using a six-variable compositional space, 397 unique copolymer compositions were synthesized of which > 10 outperformed state-of-the-art materials, demonstrating such an approach to be efficient at tackling high-dimensional structure–property relationships that are otherwise difficult to model. Moreover, the discovery of new polymer designs for flame retardants was explored using a ML-assisted approach while manually synthesizing the proposed polymers [34].

## Analytical Methods Developed and Enhanced by Machine Learning

### Chromatography

#### Chromatographic Response Functions Are Needed to Drive ML Algorithms

There are many ML approaches developed already that are capable of aiding in the development of LC methods [8, 35, 36]. However, incorporating them in a closed-loop fashion remains challenging but has been demonstrated in a few examples using a Bayesian optimization algorithm [37–39]. The prime bottleneck appears to be the development of a chromatographic response function (CRF) that can guide the optimization process.

The above Bayesian optimization approaches were demonstrated using small molecules. Polymer characterization through LC often does not yield single peaks to be separated, but rather distributions. Defining a CRF that a ML algorithm can optimize is therefore more challenging. The simplest way to define such a function is by trying to achieve a resolution of at least 1.5 between neighboring peaks. While this may not be applicable to classical synthetic polymers, it has been investigated for oligonucleotides [40]. The authors demonstrated the use of a support vector regression (SVR) model to predict the resolution between impurities in 12- and 16-mer oligonucleotide sequences for ion-pair reversed-phase LC. This model may be used to aid in predicting suitable method conditions for arbitrary sequences [40].

Nevertheless, the development of specialized CRFs tailored to distribution analysis would be highly beneficial. There appear to be at least two potential strategies for such a CRF. [41]. The first strategy would aim to enhance resolution within a single distribution by stretching it as much as possible, providing more detailed insights into the resolved distribution. However, this approach has practical limitations as excessive stretching can result in a distribution that is too wide, leading to low signal intensity and poor detection at the outer edges of the distribution. The second strategy would focus on maximizing the separation between multiple distributions. To incorporate these strategies into CRFs and enable automated optimization for distribution separation, it is essential to accurately characterize the distributions. The simplest method for this involves characterizing distributions using their average moments, such as mean retention/elution time, asymmetry, and kurtosis.

#### Prediction of Polymer Solubility to Optimize Separations

An intriguing property of synthetic polymers is their ability, or inability, to dissolve in specific solvents. This characteristic is particularly critical in techniques such as liquid chromatography where the analyte must be soluble in at least the strong solvent used during the process. Accurately predicting the solubility of novel polymers can be challenging. However, modeling and predicting this behavior could provide significant advantages in both research and application contexts. ML approaches can be employed to estimate the Flory–Huggins interaction parameter of a polymer–solvent mixture [42–44], which provides valuable insights into solubility. Alternatively, neural networks can be trained using large databases that include solvent compatibility information for various polymers. By leveraging polymer structural data, these models can predict suitable solvents with a high degree of accuracy [45, 46].

While these solubility models have not yet been applied to the optimization of chromatographic separations, they do offer a potential solution for automated method optimization. Common chromatographic modes for synthetic polymers rely on polymer precipitation and subsequently redissolving for elution. This is performed using either a solvent gradient, which is referred to as gradient polymer-elution chromatography (GPEC), or a temperature gradient, referred to as temperature gradient interaction chromatography (TGIC). Moreover, to perform LC under critical conditions, a combination of a “good” and “bad” solvent is required in a specific ratio to obtain a mass-independent separation condition. While these critical solvent conditions are known for a handful of polymers, it would be a great advantage if such conditions could be predicted by solubility models.

### Detection and Identification Methods

#### Detection

 While significant progress has been made in applying ML to method development on the chromatographic side, advancements in detection techniques and the analysis of resulting polymer data have been comparatively limited. Frequently, dedicated algorithms based on expert knowledge are employed to extract specific information from the data [47–49]. For ML to be effectively utilized in this domain, it is crucial to generalize problems across multiple polymer types. Nevertheless, ML has demonstrated its utility in efficiently solving complex problems, particularly in fitting intricate models to challenging datasets. An example of this is the determination of block length distributions in copolymers based on fragment data [50].

#### Identification

Machine learning can be highly advantageous for processing raw data and translating it into more informative insights. A common application is the identification of classes within datasets, typically achieved by training a model on labeled data where the class distinctions are known. For instance, ML has been employed to identify microplastics [51]. In one study, a random forest model was trained to distinguish between polymethyl methacrylate (PMMA), polystyrene (PS), polytetrafluoroethylene (PTFE), polyvinylchloride (PVC), and polyethylene (PE) based on Raman spectra obtained from environmental samples [52]. Similarly, leveraging infrared (IR) spectroscopy data, classification techniques have been used to identify plasticizers in PVC [53] and nylon particles [54].

The same principles can be extended to mass spectrometry (MS) data, including imaging MS [55]. While these identification methods typically determine the most likely class, they often provide limited insights into the probability of the classification. Logistic regression is frequently employed for such tasks, and when threshold criteria are omitted, it can also be used to report probabilities for various classes. This approach has been applied, for instance, to identify monomers leaching from dental composites [56]. ML has also been employed to discriminate between virgin and recycled poly(ethylene terephthalate) (PET) based on data from headspace comprehensive two-dimensional gas chromatography coupled with mass spectrometry [57].

Beyond direct identification, ML is also applied to predict polymer characteristics such as their size. These predictions can assist in identification processes, particularly with techniques like ion mobility spectrometry where ML is used to predict the collision cross-section of polymers. Such predictions help narrow the search window for potential candidates, enhancing the efficiency and accuracy of the identification process [58].

## Future Perspectives

**Fig. 1:**
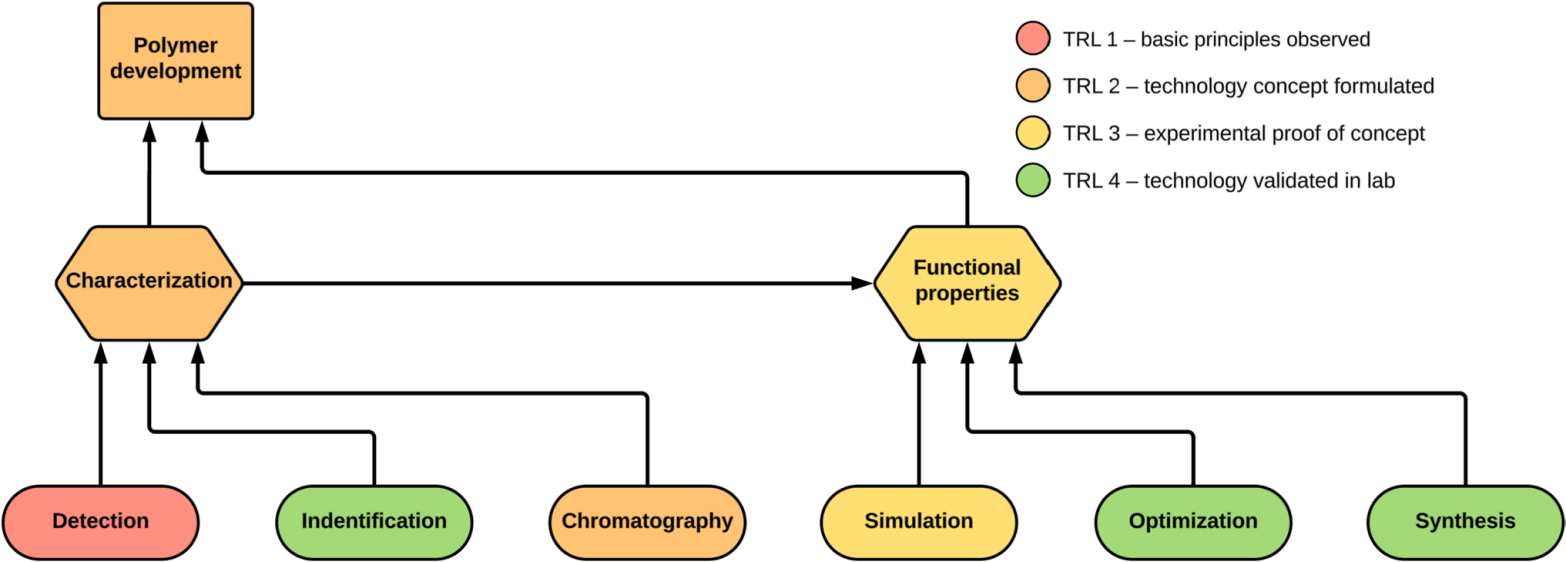
Flowchart illustrating the various aspects of polymer development that can be enhanced by optimizing functional properties through polymer characterization. The colors represent the current technology readiness level (TRL) of machine learning applicability in each aspect of the application for polymers as defined by the European Commission in the Horizon [59]

ML is increasingly applied across various aspects of polymer development and characterization, accelerating research and expanding capacity. While ML often enhances a single component of the workflow, its potential extends to multiple stages of the process. Although ML has demonstrated success in optimizing synthesis procedures to improve polymer properties and classifying polymers using established methods, greater attention should be directed toward automating method development for characterization techniques as well as improving data processing and interpretation. This shows that the readiness of different aspects of polymer science to benefit from ML varies significantly. These varying levels of readiness are depicted in Fig. 1. Addressing the less-developed areas is essential for achieving a fully versatile and integrated workflow. The primary areas with significant potential for advancement are detection techniques, chromatography, and the analysis of the resulting data. Applying ML in these fields to optimize methods offers the potential to greatly enhance sensitivity and separation performance. Furthermore, ML-based models for data interpretation can uncover novel insights into structure–property relationships that were previously inaccessible.

Advancements in ML are expected to accelerate the optimization process, thereby reducing the number of steps and measurements required in experimental workflows. Another promising avenue is the integration of ML with expert knowledge of fundamental principles. This can be achieved by supplementing experimental data with data from simulations. A recent example of an algorithm capable of handling this approach is multi-task Bayesian optimization [37]. These algorithms can identify and correct biases in simulations based on experimental data, enabling efficient optimization of more complex problems.

A distinctly different, yet rapidly advancing class, of algorithms is large language models (LLMs). LLMs are fundamentally trained to generate and predict patterns based on vast amounts of data. They "mimic" understanding rather than develop a mechanistic model of the process being optimized. While LLMs can suggest optimizations based on learned patterns, they do not explicitly model uncertainty or actively search for the optimal solution in a structured way. While LLMs are unlikely to contribute directly to optimization or characterization processes, since they do not inherently understand data but rather mimic understanding, they hold potential in facilitating more intuitive interactions with laboratory equipment and specialized data processing software. Additionally, they could play a valuable role in streamlining reporting and documentation processes, enhancing accessibility and efficiency in polymer research.

We envision a future where these advancements empower researchers to dedicate more time to addressing scientific questions and interpreting results while also enhancing and streamlining method development and synthesis processes. While this level of automation may require years or decades to achieve, the eventual integration of laboratory processes could lead to the creation of interfaces capable of recommending experiments, within the laboratory’s capabilities, to effectively answer specific research questions.

## Data Availability

No datasets were generated or analysed during the current study.
